# The influence of social media on recruitment to surgical trials

**DOI:** 10.1186/s12874-020-01072-1

**Published:** 2020-07-28

**Authors:** Carly Nichola Bisset, Ben Carter, Jennifer Law, Jonathan Hewitt, Kat Parmar, Susan Joan Moug, on behalf of the ELF Study Group Collaborative Authorship, Bryony Ross, Julia Oleksiewicz, Nicola Fearnhead, Christopher Jump, Jemma Boyle, Alex Shaw, Jonathan Barker, Jane Hughes, Jonathan Randall, Isileli Tonga, James Kynaston, Matthew Boal, Nicola Eardley, Elizabeth Kane, Harriet Reader, Sunanda Roy Mahapatra, Michael Garner-Jones, Jessica Juliana Tan, Said Mohamed, Rina George, Ed Whiteman, Kamran Malik, Christopher J. Smart, Monica Bogdan, Madhu Parna Chaudhury, Videha Sharma, Daren Subar, Panna Patel, Sok-Moi Chok, Evelyn Lim, Vedamurthy Adhiyaman, Glesni Davies, Ellen Ross, Rudra Maitra, Colin W. Steele, Campbell Roxburgh, Shelly Griffiths, Natalie S. Blencowe, Emily N. Kirkham, John S. Abraham, Kirsty Griffiths, Yasser Abdulaal, Muhammad Rafaih Iqbal, Munir Tarazi, James Hill, Azam Khan, Ian Farrell, Gemma Conn, Jugal Patel, Hyder Reddy, Janahan Sarveswaran, Lakshmanan Arunachalam, Afaq Malik, Luca Ponchietti, Krystian Pawelec, Yan Mei Goh, Parveen Vitish-Sharma, Ahmed Saad, Edward Smyth, Amy Crees, Louise Merker, Nahida Bashir, Gethin Williams, Jennifer Hayes, Kelly Walters, Rhiannon Harries, Rahulpreet Singh, Nikola A. Henderson, Francesco M. Polignano, Ben Knight, Louise Alder, Alexandra Kenchington, Yan Li Goh, Ilaria Dicurzio, Ewen Griffiths, Ahmed Alani, Katrina Knight, Patrick MacGoey, Guat Shi Ng, Naomi Mackenzie, Ishaan Maitra, Susan Moug, Kelly Ong, Daniel McGrath, Emanuele Gammeri, Guillame Lafaurie, Gemma Faulkner, Gabriele Di Benedetto, Julia McGovern, Bharathi Subramanian, Sunil Kumar Narang, Jennifer Nowers, Neil J. Smart, Ian R. Daniels, Massimo Varcada, Tanzeela Gala, Julie Cornish, Zoe Barber, Stephen O’Neill, Richard McGregor, Andrew G. Robertson, Simon Paterson-Brown, Thomas Raymond, Mohamed A. Thaha, William J. English, Cillian T. Forde, Heidi Paine, Alpa Morawala, Ravindra Date, Patrick Casey, Thomas Bolton, Xuan Gleaves, Joshua Fasuyi, Sanja Durakovic, Matt Dunstan, Sophie Allen, Angela Riga, Jonathan Epstein, Lyndsay Pearce, Emily Gaines, Anthony Howe, Halima Choonara, Ffion Dewi, Joanne Bennett, Emile King, Kathryn McCarthy, Greg Taylor, Dean Harris, Hari Nageswaran, Amy Stimpson, Kamran Siddiqui, Lay In Lim, Christopher Ray, Laura Smith, Gillian McColl, Mohammed Rahman, Aaron Kler, Abhi Sharma, Kat Parmar, Neil Patel, Perry Crofts, Claudio Baldari, Rhys Thomas, Michael Stechman, Roland Aldridge, James O’Kelly, Graeme Wilson, Nicholas Gallegos, Ramya Kalaiselvan, Rajasundaram Rajaganeshan, Aliya Mackenzie, Prashant Naik, Kaushiki Singh, Harinath Gandraspulli, Jeremy Wilson, Kate Hancorn, Amir Khawaja, Felix Nicholas, Thomas Marks, Cameron Abbott, Susan Chandler

**Affiliations:** 1 Department of General Surgery, Royal Alexandra Hospital, Paisley, PA2 9PN UK; 2 Department of Biostatistics and Health Informatics, Institute of Psychiatry, Psychology & Neuroscience, King’s College London, London, UK; 3 Department of General Surgery, Blackpool Victoria Hospital, Blackpool, UK; 4 Department of Population Medicine, Cardiff University, Cardiff, UK; 5 Department of General Surgery, Manchester Royal Infirmary, Manchester, UK

**Keywords:** Social media, Recruitment, Surgical trials

## Abstract

**Background:**

Social media has changed the way surgeons communicate worldwide, particularly in dissemination of trial results. However, it is unclear if social media could be used in recruitment to surgical trials. This study aimed to investigate the influence of Twitter in promoting surgical recruitment in The Emergency Laparotomy and Frailty (ELF) Study.

**Methods:**

The ELF Study was a UK-based, prospective, observational cohort that aimed to assess the influence of frailty on 90-day mortality in older adults undergoing emergency surgery. A power calculation required 500 patients to be recruited to detect a 10% change in mortality associated with frailty. A 12-week recruitment period was selected, calculated from information submitted by participating hospitals and the numbers of emergency surgeries performed in adults aged > 65 years. A Twitter handle was designed (@ELFStudy) with eye-catching logos to encourage enrolment and inform the public and clinicians involved in the study. Twitter Analytics and Twitonomy (Digonomy Pty Ltd) were used to analyse user engagement in relation to patient recruitment.

**Results:**

After 90 days of data collection, 49 sites from Scotland, England and Wales recruited 952 consecutive patients undergoing emergency laparotomy, with data logged into a database created on REDCap. Target recruitment (*n* = 500) was achieved by week 11.

A total of 591 tweets were published by @ELFStudy since its conception, making 218,136 impressions at time of writing. The number of impressions (number of times users see a particular tweet) prior to March 20th 2017 (study commencement date) was 23,335 (343.2 per tweet), compared to the recruitment period with 114,314 impressions (256.3 per tweet), ending June 20th 2017. Each additional tweet was associated with an increase in recruitment of 1.66 (95%CI 1.36 to 1.97; *p* < 0.001).

**Conclusion:**

The ELF Study over-recruited by nearly 100%, reaching over 200,000 people across the U.K. Branding enhanced tweet aesthetics and helped increase tweet engagement to stimulate discussion and healthy competition amongst clinicians to aid trial recruitment. Other studies may draw from the social media experiences of the ELF Study to optimise collaboration amongst researchers.

**Trial registration:**

This study is registered online at www.clinicaltrials.gov (registration number NCT02952430) and has been approved by the National Health Service Research Ethics Committee.

## Background

Social media is the term used to describe the interactive, web-based free applications, that have been instrumental in increasing accessibility of information by allowing instantaneous worldwide communication. The reach of social media in modern society is extensive, with an estimated 2.46 billion users of any social media application reported in 2017 [1].

One of these applications is Twitter (Twitter Inc., San Francisco, CA, USA): an online micro-blogging site which limits messages (or ‘tweets’) to 280 characters or less, and may be attached to an image or website link and can connect to other notable Twitter users/institutions by ‘tagging’ and thereby including other users. Twitter is an ideal social media modality to convey concise messages on a public platform, is free at point of service and is often used professionally to represent the views of that user or institution. Social media usage is often associated with increasing engagement with professional bodies and surgical societies such as the Association of Coloproctologists’ of Great Britain and Ireland (ACPGBI) [2]. There is also some evidence to suggest that the age of the user will affect platform preference in social media usage, for example amongst emergency medicine physicians, trainees tended to use Facebook and YouTube, whilst older, more experienced physicians used Twitter and LinkedIn [3]. Whether or not this is true of general surgery is yet to be established.

In surgery, the role of social media is evolving, with reported beneficial effects including: facilitating patient education, sharing information on new guidelines or published research and increasing collaboration amongst stakeholders such as: patients, clinicians, industry, trainees and educational institutions [4, 5].

These benefits to research raise the question of the influence of social media on performing research, in particular: recruitment to trials. It is anticipated that recruitment to research trials using social media may substantially increase recruitment numbers in a cost-effective manner [6]. Little is known in this area, however social media outlets such as Twitter could facilitate recruitment specifically to surgical trials and engagement in research, such as that seen in the National Audit of Small Bowel Obstruction study (NASBO) [7]. This study demonstrated that tweets which involved images and tagged other users, led to higher levels of engagement with tweets and maintained collaborator engagement. Unfortunately within surgical research, use of social media is variable amongst different specialties in the UK including: colorectal surgeons (3.1%) [2], vascular surgeons (4.8%) [7] and plastic surgeons (22%) [8], which overall seems low in comparison to the estimated 1 in 5 doctors in the UK using Twitter on a daily basis [9].

The Emergency Laparotomy and Frailty (ELF) Study was a multi-disciplinary, multicentre prospective cohort study undertaken in the UK in 2017 [10]. Using Twitter (@ElfStudy) to advertise for site registration and patient recruitment, the ELF Study achieved its target recruitment early, reporting significant over-recruitment at completion. This study considers the influence of social media on ELF Study’s recruitment success, offering insight on how clinical trial groups may use social media-based branding strategies to promote recruitment to surgical trials.

## Methods

The ELF Study investigated the relationship to pre-operative frailty and mortality at 90-days in older adults undergoing emergency laparotomy in the U.K. (trial registration number NCT02952430). Consecutive patient recruitment was performed for 3 months (20th March 2017 - 20th June 2017) with each patient subsequently followed up for 90-days. Target recruitment was calculated with 500 patients anticipated to detect a 10% change in mortality associated with frailty. Ethical approval was given by the National Health Service Research Ethics Committee (Black Country Research Committee, November 2016; 16/WM/0500), with central registration at the Health Research Authority (HRA) for English sites, NHS Research Scotland Permissions Coordinating Centre (NRSPCC) for Scottish sites and Health and Care Research Permissions Service for Welsh sites [11]. Patient consent was not necessary for this study.

The ELF Study was a trainee-led initiative, led by the North West Research Collaborative (surgical trainees) and the Older Persons Surgical Outcomes Collaboration (OPSOC: surgeons and geriatricians). The principal investigators at each site were either general surgery registrars or consultant surgeons in a hospital performing emergency abdominal surgery and peri-operative care. Principal investigators led local teams of trainees, but each team required a consultant general surgeon to ensure collaborators acted in accordance with local clinical governance and guidelines.

The eligibility criteria for the ELF Study is listed below:

- Patients aged > 65 years
- Patients who undergo an expedited, urgent or emergency abdominal procedure on the gastrointestinal tract, including:

-  ○ Open, laparoscopic or laparoscopic-assisted procedures
-  ○ Procedures involving the stomach, small or large bowel, or rectum for conditions such as: perforation, ischaemia, abdominal abscess, bleeding or obstruction
-  ○ Washout/evacuation of intraperitoneal haematoma or abscess (except when due to appendicitis or cholecystitis)
-  ○ Bowel resection/repair due to incarcerated umbilical, inguinal and femoral hernias, or acute incisional hernias
-  ○ Laparotomy or laparoscopy with inoperable pathology (i.e. peritoneal or hepatic metastases)
-  ○ Laparoscopic/open adhesiolysis
-  ○ Return to theatre for repair of substantial dehiscence of major abdominal wound (i.e. ‘burst abdomen’), or any major post-operative complication

The Twitter handle @ElfStudy was opened (8th January 2017) with eye-catching and consistent ‘branding’ used to advertise for site registration prior to the recruitment period (10 weeks prior to the date of study commencement). This branding took the form of a catchy and consistent colour scheme (red and green), and used a study-specific Twitter account (@ELFStudy). This Twitter account provided regular public updates on: study recruitment requirements, news on data collection sparking regional and hospital-based light-hearted competition, sharing links to related research articles of interests and presentations by the steering group on provisional ELF Study results at general surgery, geriatrics and anaesthetic research meetings.

### Definitions

Twitter Analytics and an online analytics tool (Twitonomy; Digonomy Pty Ltd) were used to examine potential patterns or changes in user engagement in relation to weekly progression of the study, with definitions described in Table 1.

**Table 1:** Definitions used in Twitter Analytics

Analytical Tool	Definition
Impressions	Number of times users see a particular tweetTotal number of times a tweet was seen by individual users on Twitter
Engagements	Total number of times a tweet was interacted with (including users opening a link, re-tweeting, replies to tweets, ‘likes’)
Re-tweet	The forwarding of an original tweet to another user’s profile, sharing information with other users
Likes	Users indicate they agree with the sentiment of tweet content
Followers	Number of users who receive updates on a particular account’s posts (i.e. the greater number of followers, the greater the likelihood of higher tweet engagement)
Tags / Tagging	Including another user in Tweet content and/or directing a message at another user, often increasing the engagement of the Tweet e.g. @ELFStudy
Hashtags	‘Tagging’ groups a collection of tweets with similar content so that users may find a common thread of tweets on a similar topic e.g. #colorectalsurgery, #surgicaltraining
User	An individual or an organisation with a Twitter account
Handle	The username beginning with “@” which is unique to that Twitter account e.g. @ELFStudy

### Outcome and exposure

Exposure of social media was defined in three ways: the total number of tweets by @ELFStudy; the total number of the impressions and engagements of each tweet (a marker of the reach of each tweet and interactions); and the maximum number of impressions and engagements with a single tweet in the week. Cumulative recruitment (i.e. weekly recruitment of patients) and number of tweets were our primary measures of outcome and exposure. The final week was excluded (week 13 - study end date), as some sites had collated their recruitment numbers and reported them in Week 13 to a database set up using REDCap, rather than contemporaneously over the course of the trial. The overall denominator for the number of recruiting surgeons and trainees cannot be calculated using a social media approach, as the number of impressions may include irrelevant individuals, groups or organisations.

The pre-trial phase and recruitment phase were selected for comparison as the content of tweets changed between the two periods. In the pre-trial phase, most tweet content was based upon individual sites signing up to the trial for patient recruitment, with general information on how to become involved in the ELF Study. In the recruitment phase, tweet content became more focussed on maintaining recruitment momentum, with site-specific and whole-study updates which encouraged competition. Tweets from the recruitment phase included: site league tables, photographs from local recruiters (for example, containing ‘elf’ toys facilitating the branding strategy) and general trial progress updates.

### Statistical analysis

**Fig. 1:**
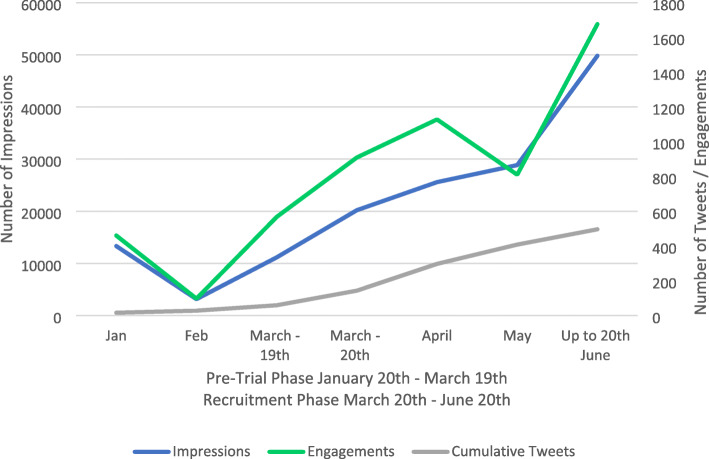
Comparison of Impressions and Engagements between Phase 1 (pre-trial) and Phase 2 (patient recruitment)

Tweets published by @ELFStudy were divided into two time phases to allow comparison to weekly recruitment figures: 1) pre-trial commencement (pre-trial phase), 2) trial commencement and data collection (recruitment phase – date selected for commencement March 20th 2017 and date of first patient recruited). Tweets after these time phases were excluded.

For the 12 weeks of the study, we fitted a linear regression to model cumulative recruitment. To assess the additional impact of the social media campaign we fitted: sum of engagement; and total cumulative number of tweets as two independent analyses. The different models were compared using the adjusted coefficient to determination (adjusted r^2^) summary. The slope parameter was presented alongside the 95% Confidence interval and *p*-value.

## Results

### Study recruitment

A total of 52 sites registered with 49 submitting data to a database set up in REDCap. There was a total of 161 collaborators (54 consultants, 107 trainees). The target recruitment (*n* = 500) was achieved by week 11 with recruitment increasing exponentially from week 11 to almost double the intended target by week 12 (*n* = 952) (Table 2). A total of 926 tweets were published by @ELFStudy during the pre-trial (phase 1) and recruitment phases (phase 2). Site recruitment of patients meeting the inclusion criteria was performed by junior doctors and consultants. Interested users on Twitter could choose to ‘follow’ the @ELFStudy account, which is open to the public, to receive updates on the study.

**Table 2:** Weekly recruitment comparison with tweet engagement

Week	Weekly recruitment	Weekly number of tweets	Weekly Total Impressions	Weekly Total Engagements	Cumulative Patient Recruitment	Cumulative number of tweets
1	30	30	7938	553	30	30
2	28	55	8142	384	58	85
3	41	61	11,261	470	99	146
4	23	8	1027	18	122	154
5	49	56	8033	448	171	210
6	56	20	3960	164	227	230
7	67	33	2528	50	294	263
8	69	23	4254	103	363	286
9	45	30	12,020	426	408	316
10	62	20	7459	182	470	336
11	37	15	6780	160	507	351
12	100	35	12,979	552	607	386

### Impressions and engagements

**Fig. 2:**
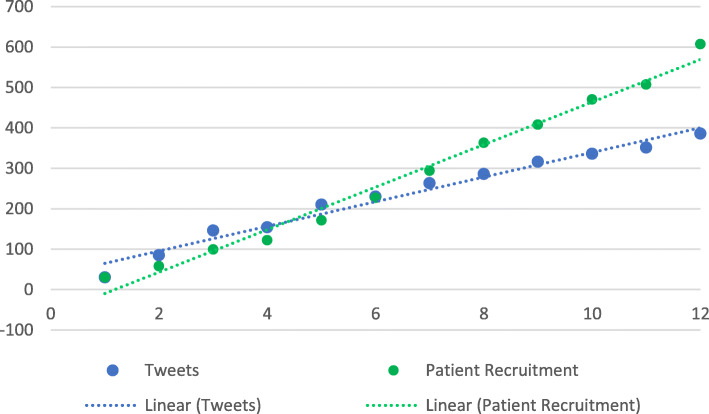
Cumulative Weekly Tweets & Patient Recruitment

The number of phase 1 tweets was 69, resulting in 23,335 impressions (343.2 per tweet) versus 437 tweets published in phase 2 generating 114,314 impressions (256.3 per tweet) (Fig. 1). The number of engagements during phase 1 was 1124, compared to 4523 engagements in phase 2, with the recruitment phase seeing four times the number of tweet engagements of that seen in the pre-trial phase. This suggests that more users were reached and were more engaging with tweets published by @ELFStudy during the recruitment phase, possibly reflecting increased overall recruitment of patients (Fig. 2).

**Fig. 3:**
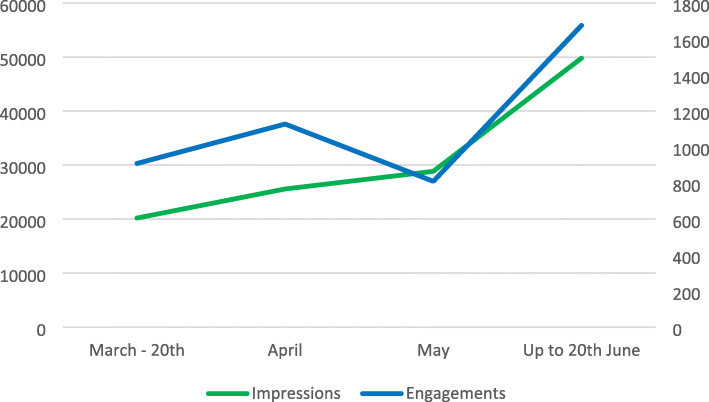
Comparison of Weekly Impressions and Engagements during Phase 2 (patient recruitment)

Week 6 marked the time period where recruitment exceeded the target prediction, however this did not bear correlation to the number of tweet engagements and impressions (Fig. 3).

Interestingly from week 11, the number of impressions and engagements markedly increased perhaps reflecting the ‘final push’ effect of individual sites logging data. This is reflected by the overall number of tweets published by @ELFStudy, which predictably had a large amount of activity at the beginning and latter stages of the recruitment phase, with a moderate number of tweets published in the mid-way point (Fig. 3).

### Data analysis

**Fig. 4:**
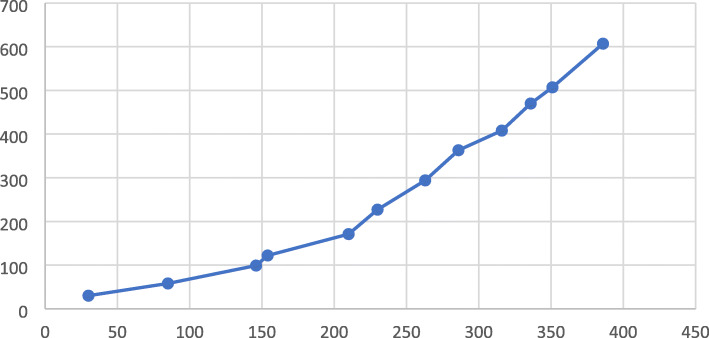
A Scatter plot of the Cumulative number of tweets with cumulative total recruitment (*r*^2^ = 0.94)

We present the aggregate results of the social media data and weekly recruitment data (Table 2). This table demonstrates a weekly temporal effect, where there is an increasing number of patients recruited on a weekly basis and increasing tweet engagement. After fitting the two linear regressions, it was clear that the cumulative number of tweets was associated with the cumulative recruitment (Fig. 4). It was found that the adjusted-*r*^2^ = 0.94. and each additional tweet was associated with an increase in recruitment of 1.66 (95% CI 1.36, to 1.97; *p* < 0.001). There was no association between the cumulative engagement and cumulative recruitment.

## Discussion

Social media has revolutionised academia and the distribution of information. Not only do social media outlets such as Twitter inform users of trial updates, it also has a potential role in changing clinical practice with trial conclusion. The vast online network connects users of the platform across the globe instantaneously to discuss a common theme and share experiences, particularly with the use of hashtags which group together messages that may be followed more easily by users who search for that hashtag item (e.g. #colorectalresearch).

Part of the successful social media recruitment drive to this study was undoubtedly due to the non-professional marketing strategy of the ELF Study, which played upon a Christmas theme in keeping with its namesake. Consistent use of a Christmas colour scheme and themed logo made the study more visible amongst its peers and was easily identifiable when presented at meetings as oral and poster presentations (Additional Figures 1 and 2). This reflects standardised marketing strategies by other major industries which recognise that consumer participation increases engagement quality and experiences (https://sysomos.com/inside-twitter/twitter-statistics/ (accessed 28/2/19)). A professional marketing strategy was not considered due to high anticipated costs, within a small funding budget from the Bowel Disease Research Fund (charitable status). Twitter provides a free at point-of-access online platform which has a large colorectal surgeon and trainee membership, and users can possess multiple accounts (e.g. a personal account, and an organisation account such as @ELFStudy) [2]. With increasing popularity of Twitter profiles for research trials, it is becoming more difficult to ‘stand out’ and have a visible online presence [12]. Creative strategies in advertising which engage consumers recognise: ‘play on words’ appeal (e.g. ELF), animation (e.g. use of GIFs and other illustrations) and social appeal (e.g. as part of the increasing spread of multi-centre, multi-author collaborative studies) as being highly influential factors which increase consumerism [13, 14].

The successes of the ELF Study in recruiting patients ahead of time, and in excess, may be reflected by the findings of Nowotarski et al., who found that of 179 adults recruited for a medical trial, only 30% were aware of information providing clinical trial websites (e.g. clinicaltrials.gov). Once these adults were informed of the online network available for trial recruitment, 81% showed interest in engaging with relevant clinical trials [15]. Whilst our study did not directly investigate patient information and consent to surgical trials, there is some evidence that social media may facilitate the education of patients and clinicians on relevant clinical trials. The open manner with which information is received through social media may increase engagement, as users have a direct contact (or a ‘face’) they can seek further information from. Site recruitment will also have been facilitated by ‘word-of-mouth’ marketing, via presentations and networking at surgical and geriatric medicine conferences. The open nature of social media is further enhanced by collaboration and sharing of information (including sharing of presentations at conferences), and allows multi-centre studies to communicate with individual sites and collectively with all sites to address problems with trial protocol, trial recruitment and to instil a sense of ‘healthy competition’, where sites could compete for optimal trial recruitment and participation.

Twitter was the only social media modality used for the ELF Study and this paper, given the high level of professional usage within the outlet, in comparison to other platforms which have not been examined as part of this study, which many users have for personal and not professional reasons [2]. As is true with all social media modalities which are instantaneous, in comparison to traditional industrial media (e.g. printed news, television), communications such as tweets may be altered at any time and ‘deleted’, meaning that social media data may be viewed as less ‘permanent’ than industrial level media [16]. In order to ethically manage this and moderate pages, several boards have drawn consensus on moderating the use of social media platforms for professional purposes such as the General Medical Council (UK). There is increasing governance over content of social media posts in medicine, particularly relating to patient confidentiality, or other violations of professionalism such as: inappropriate contact with patients, declaring conflicts of interest and maintaining professionalism amongst colleagues and other users [17, 18].

As recruitment data was collected per week, daily analysis of the effect of tweets on patient recruitment was not possible. It is likely that the large number of impressions @ELFStudy generated (a surrogate marker for study ‘reach’) included individuals and organisations who may not have an interest in peri-operative patient care. The number of impressions may also be higher than the actual number of users seeing an individual tweet, particularly where users have multiple accounts (for example, a personal and an organisation account). It is not currently possible to identify which users met the intended ‘target audience’ for recruitment, or which users contribute to tweet impressions. However, it is also possible that Twitter users in our group share an ‘echo chamber’; where Twitter updates are seen and shared by like-minded users, contributing to confirmation bias.

## Conclusion

The ELF Study is a multi-disciplinary, multi-centre trial whose success was undoubtedly assisted by social media and non-professional marketing. Eye-catching logos and consistent colour schemes enhanced tweet aesthetics and helped increase tweet engagement to stimulate discussion and healthy competition amongst clinicians to aid trial recruitment.

Social media usage in surgical research is becoming standard, however its role in other medical specialties is still to be explored and utilised; including geriatric medicine where there is no current evidence to demonstrate clinician engagement and patterns of usage. The ELF Study achieved target recruitment within half of the predicted time period, a feat not to be underestimated. Other studies may draw from the social media experiences of the ELF Study, including: regular updates to inform participants of study progress, successful ‘branding’ playing on the study acronym and ultimately engaging a multi-disciplinary platform of: physicians, surgeons and anaesthetists to collaborate and produce high quality peri-operative outcomes data.

## Supplementary information

**Additional file 1: Figure S1.** The Elf Study logo as an example of the branding strategy. **Figure S2.** Example of tweet media content using the branding strategy

## Data Availability

The datasets used and/or analysed during the current study are available via Twitter Analytics and Twitonomy, or from the corresponding author on reasonable request.

## Abbreviations

ELF: Emergency Laparotomy and Frailty Study
ACPGBI: Association of Coloproctology of Great Britain and Ireland
NASBO: National Audit of Small Bowel Obstruction study
HRA: Health Research Authority
NRSPCC: NHS Research Scotland Permissions Coordinating Centre
