# Supplementary material for: The influence of social media on recruitment to surgical trials
Source: BMC Med Res Methodol. 2020 Jul 28;20:201. doi: 10.1186/s12874-020-01072-1 (PMC7388470; doi:10.1186/s12874-020-01072-1)

Additional Figure 1:

The Elf Study logo as an example of the branding strategy


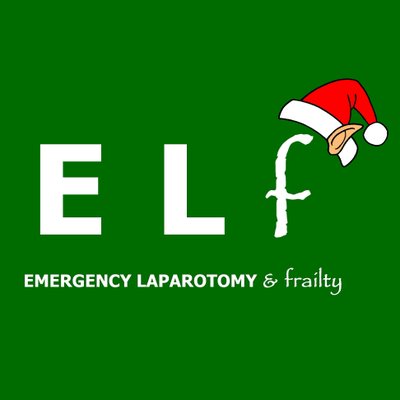


Additional Figure 2: Example of tweet media content using the branding strategy


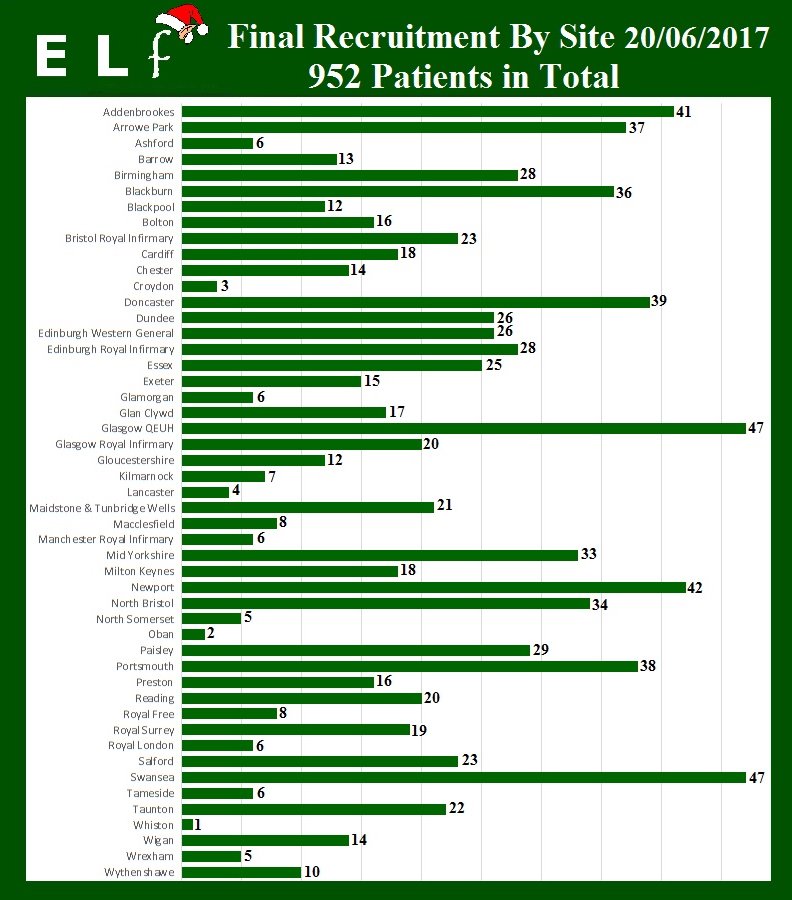

Supplement: Supplementary file 1 — Additional file 1: Figure S1. The Elf Study logo as an example of the branding strategy. Figure S2. Example of tweet media content using the branding strategy [file 12874_2020_1072_MOESM1_ESM.docx]
